# 1212. Impact of Infectious Diseases Fellow Collaboration in the Medical Intensive Care Unit and Medical Intermediate Unit on Antimicrobial Utilization in COVID-19 Patients

**DOI:** 10.1093/ofid/ofad500.1052

**Published:** 2023-11-27

**Authors:** Smit Rajput, Franklin Mikell, Aaron Kipp, Jennifer Emberger

**Affiliations:** East Carolina University, Greenville, North Carolina; ECU Health Brody School of Medicine, Greenville, North Carolina; East Carolina University Brody School of Medicine, Greenville, North Carolina; East Carolina University Health Medical Center/Brody School of Medicine, Greenville, North Carolina

## Abstract

**Background:**

During the COVID-19 pandemic, historic gains made in antibiotic stewardship were challenged as antibiotic utilization for patients with COVID-19 increased despite low co-infection rates, as evidenced by a 15% increase in resistant infections 2019 - 2020. Studies demonstrate that input of an infectious diseases (ID) specialist can improve appropriate antibiotic use without increasing length of stay or mortality. But, there is limited data on this among COVID-19 patients. We evaluated the impact of an ID fellow rounding in the Medical ICU (MICU) and medical intermediate unit (MIU) on antibiotic prescribing among COVID-19 patients admitted to a tertiary care center in eastern North Carolina.

**Methods:**

An ID fellow rounded with the MICU and MIU teams twice weekly to provide antibiotic guidance from 3/21-6/21. The intervention group comprised patients admitted with COVID-19, prescribed antibiotics, and receiving antibiotic guidance. Concurrent (3/21 - 6/21) and retrospective (11/20 – 1/21) controls met the same criteria but without ID fellow antibiotic guidance. Antibiotic and other data were obtained from chart review. Prescribing rates (antibiotic days per 1000 hospital days) were calculated and compared using Poisson regression to estimate rate ratios while controlling for covariates.

**Results:**

Analysis included 48 intervention patients, and 38 concurrent and 64 retrospective controls. The intervention group had higher BMI, longer hospital stay, and more frequent ICU stay than either control group (Table 1). The median prescribing rate in the intervention group (788.9 antibiotic days per 1000 hospital days) was lower than the retrospective controls but higher than the concurrent controls (Figure 1). Upon adjusting for covariates, the intervention group had a 32% lower antibiotic prescribing rate than retrospective controls (rate ratio: 0.68; 95% CI: 0.50 – 0.77). A similar result was seen when compared to the concurrent controls (Table 2).

**Table 1**

**Figure:**
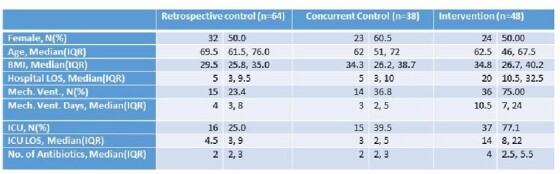

Demographic, clinical, and hospital stay characteristics, by intervention group, among those prescribed any antibiotics

**Figure 1**

**Figure:**
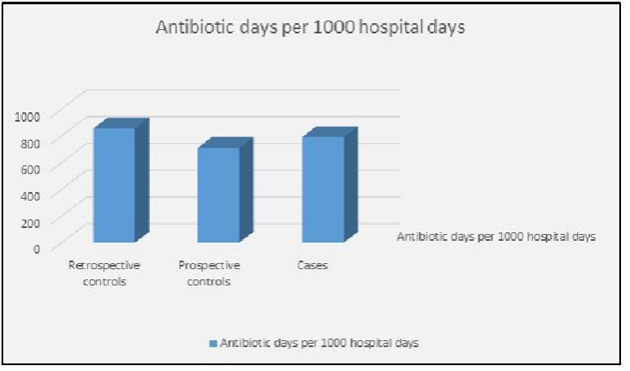

Median antibiotic days per 1,000 hospital days, by intervention group

**Table 2**

**Figure:**
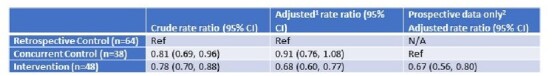

Adjusted analysis of antibiotic prescribing rate (per hospital stay) 1: Adjusted for age, BMI, and spending time in the ICU (Yes/No) 2: Restricted to only the Concurrent Control and Intervention group, Adjusted for age, BMI, and spending time in the ICU (Yes/No)

**Conclusion:**

We found a significant reduction in antibiotic prescribing among COVID-19 patients when an ID fellow provided antibiotic guidance. Our study contributes to the growing body of data that ID providers are critical to the fight against antimicrobial resistance and are valuable resources in determining the need for antimicrobials.

**Disclosures:**

**All Authors**: No reported disclosures

